# Supplementary material for: Live cell dynamics of production, explosive release and killing activity of phage tail-like weapons for Pseudomonas kin exclusion
Source: Commun Biol. 2021 Jan 19;4:87. doi: 10.1038/s42003-020-01581-1 (PMC7815802; doi:10.1038/s42003-020-01581-1)
Supplement: Supplementary file 2 — Supplementary Information [file 42003_2020_1581_MOESM2_ESM.pdf]

SUPPLEMENTARY INFORMATION

Live cell dynamics of production, explosive release and killing activity of phage tail-like weapons for *Pseudomonas* kin exclusion

Jordan Vacheron[1†]*, Clara Margot Heiman[1†], Christoph Keel[1]*
[1] Department of Fundamental Microbiology, University of Lausanne, CH-1015 Lausanne, Switzerland*

Corresponding authors: Christoph Keel (email: christoph.keel@unil.ch) and Jordan Vacheron (email: jordan.vacheron@unil.ch), Department of Fundamental Microbiology, University of Lausanne, Biophore Building, CH-1015 Lausanne, Switzerland.

Supplementary Methods
Construction of *Pseudomonas protegens* CHA0 deletion and gene replacement mutants.

Supplementary Figures
Supplementary Fig. 1. *Pseudomonas protegens* CHA0 and derivatives growth kinetics (**a**) and sensitivity to mitomycin C (MMC) (**b, c, d**).

Supplementary Fig. 2. The fluorescent label impacts the conformation of tailocin #1 and thus its efficiency.

Supplementary Fig. 3. Mitomycin C (MMC) increases the induction of tailocins more than 80-fold.

Supplementary Fig. 4. Tailocin #1 contributes to interbacterial competitiveness of *Pseudomonas protegens* CHA0 against *Pseudomonas protegens* Pf-5 (following induction).

Supplementary Tables
Supplementary Table 1. *Pseudomonas chlororaphis* and *Pseudomonas protegens* subgroup strains used in this study.

Supplementary Table 2. *Pseudomonas protegens* CHA0 derivatives and *Escherichia coli* strains used in this study.

Supplementary Table 3. Inventory of the viral particles present in the *Pseudomonas protegens* CHA0 mutant strains.

Supplementary Table 4. Details of the tailocins #1 and #2 encoding genomic region in *Pseudomonas protegens* CHA0.

Supplementary Table 5. Measurement of the length of CHA0 R-tailocins.

Supplementary Table 6. Number of *P. protegens* Pf-5 cells killed by either *P. protegens* CHA0 wild – type or *P. protegens* CHA0 Δtail2ΔmyoΔsiph cells in non-induced conditions.

Supplementary Table 7. Plasmids used in this study.

Supplementary Table 8. Oligonucleotides used in this study.

References

Supplementary Methods

Construction of *Pseudomonas protegen*s CHA0 deletion and gene replacement mutants. *P. protegens* CHA0 mutant strains (Supplementary Table 2) were created using the I-SceI system with the suicide vector pEMG[1] adapted to *P. protegens* as previously described[2], with plasmids and primers listed in Supplementary Tables 7 and 8. To do so, about 300 bp to 650 bp upstream and downstream regions of the genes of interest were amplified from *P. protegens* CHA0 wild type genomic DNA using the PrimeSTAR HS DNA polymerase (Takara®) and the appropriate primers (Supplementary Table 8). The resulting PCR products were purified using the QIAquick®Gel Extraction Kit 50 (Qiagen®) and digested using the corresponding restriction enzymes. The digested fragments were purified using the QIAquick®Gel Extraction Kit 50 and then cloned into the pEMG plasmid by triple ligation using T4 DNA ligase (Promega®). Competent *Escherichia coli* S17/λpir cells were electroporated with the purified ligation products and then rescued with 1 mL of SOC medium for 1.5 h at 37 °C. The cells were plated on NA + Km^{25} + Xgal medium. White colonies were checked by colony PCR using the GoTaq®DNA Polymerase (Promega®). The plasmids were extracted using the QIAprep Spin Miniprep Kit (QIAGEN®) and then verified by sequencing. The procedure described above was used for construction of the suicide plasmids employed for the deletion mutants. The inserts that were cloned into the suicide vectors built for the 3' tag of the sheaths, were synthetized by the company IDT® (pME11087 and pME11088). All plasmids were electroporated into *E. coli* DH5α cells.

To create *P. protegens* CHA0 mutants of interest, either electroporation or conjugation using tri-parental mating were used. For electroporation, electrocompetent *P. protegens* CHA0 cells were electroporated (machine parameters: 2.5 kV; 200 Ω; 25 µF) using electroporation cuvettes (2 mm gap size, Axon Lab®) with the respective suicide plasmids (Supplementary Table 7). The cells were immediately rescued with SOC medium for 3 h at 35 °C. The cell suspensions were then plated on NA + Km^{25} plates and incubated overnight at 30 °C permitting chromosomal integration through homologous recombination. For conjugation by triparental mating, the recipient strain (*P. protegens* CHA0 or derivatives) were grown at 35 °C overnight with agitation (180 rpm). The donor strain (*E. coli* strain harboring the suicide vector) and the helper strain (*E. coli* harboring the conjugative plasmid pME497) were grown in LB at 37 °C, overnight with shaking at 180 rpm. Aliquots of 1.5 mL of each culture were centrifuged at 10,000 rpm for 1 min. Pellets were washed twice with sterile 0.9 % NaCl solution. Pellets were then resuspended and mixed together in a total volume of 500 µL of NaCl solution. This suspension containing all strains was centrifuged at 10,000 rpm for 1 min and the pellet was suspended with 100 µL of NaCl solution. This mixture was spotted onto a sterile cellulose acetate filter (0.2 µm pore site, 2.5 cm diameter, Sartorius) placed onto a NA plate and incubated for 3 h at 35 °C. Following the incubation, the bacterial growth on the filter was suspended with 1 mL of sterile 0.9 % NaCl solution. The resulting suspension was plated onto NA + Km^{25} + Cm^{10} plates and was incubated for 48 h at 30 °C. Following either one of these two methods, single colonies were grown overnight and then electroporated with the pSW-2 plasmid and rescued with 1 mL of SOC medium, incubated at 35 °C for 1.5 h and plated on NA + Gm10 plates. Kanamycin-sensitive clones were verified by PCR using the GoTaq® DNA polymerase and adequate primers (Supplementary Table 8).

Supplementary Figures

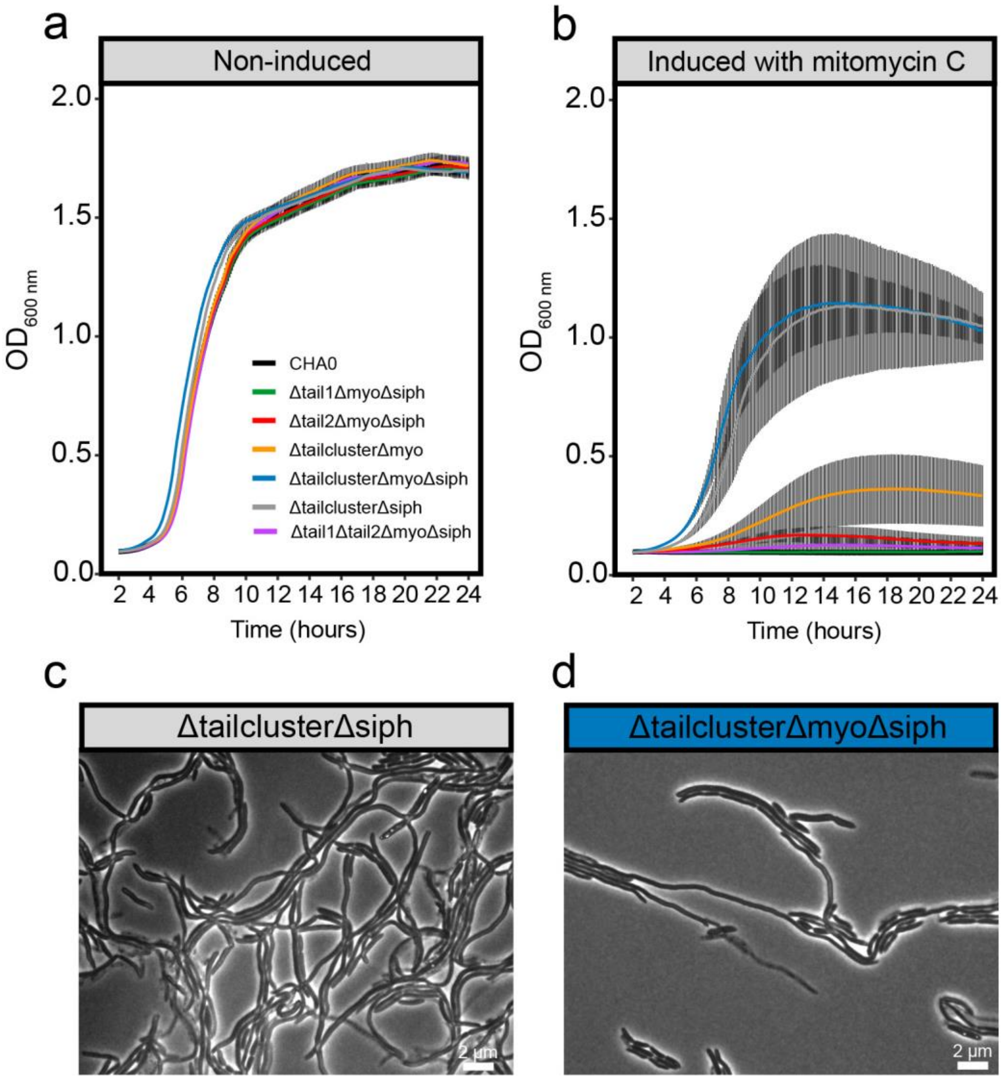

Supplementary Fig. 1. *Pseudomonas protegens* CHA0 and derivatives growth kinetics (**a**) and sensitivity to mitomycin C (MMC) (**b, c, d**). The OD600 of CHA0 wild type (black curve) and mutant derivatives (colored curves) were measured for 24 h in the absence (**a**) or presence (**b**) of 3 µg mL^{-1} of MMC to test for growth deficiencies or differential sensitivity. The mutant encoding only the myovirus sequence (ΔtailclusterΔsiph, grey curve) and the mutant no longer harboring viral sequences (ΔtailclusterΔmyoΔsiph, blue curve) appeared to be resistant to MMC as their OD600 continued to increase (**b**). However, when these mutants were visualized under the microscope, they exhibited a phenotype where cells continued elongating but no longer were able to divide due to the absence of the lytic enzymes (ΔtailclusterΔsiph and ΔtailclusterΔmyoΔsiph, respectively **c** and **d**). For the growth curves, n=5 biological independent experiments were performed. Error-bars correspond to the standard deviation.

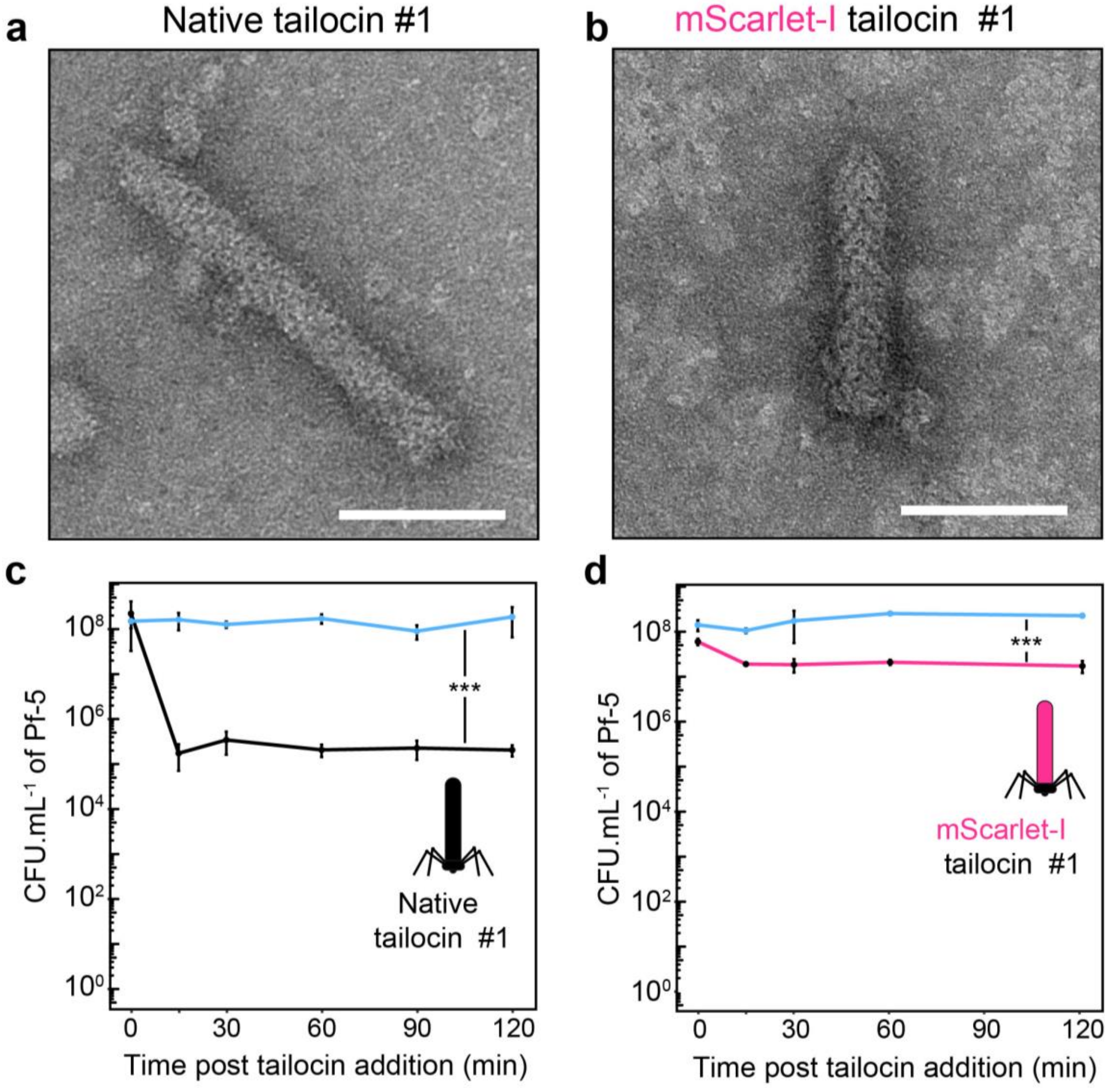

Supplementary Fig. 2. The fluorescent label impacts the conformation of tailocin #1 (**a, b**) and thus its efficiency (**c, d**). Comparison of non-tagged (**a**) and m-Scarlet-I tagged **(b)** tailocin #1 of *Pseudomonas protegens* CHA0 under transmission electron microscopy. The scale bars correspond to 100 nm. Killing activities of the purified native tailocin #1 (**c**) or the mScarlet-I-tagged tailocin #1 (**d**) of CHA0 towards *Pseudomonas protegens* Pf-5. A fresh culture of Pf-5 at exponential growth phase was faced to either Tn50 buffer (control condition, blue line) or 200 µL of purified native tailocin #1 (black line) or tagged tailocin #1 (red line). The number of Pf-5 cells was monitored by CFU counting. The experiment was performed three times independently. Student's t-test was used to detect significant differences between the control condition and the respective tailocin #1 conditions at each time point (****P* value < 0.001). Error bars correspond to the standard deviation. For the survival assay, n=3 biological independent experiments were performed. Error-bars correspond to the standard deviation.

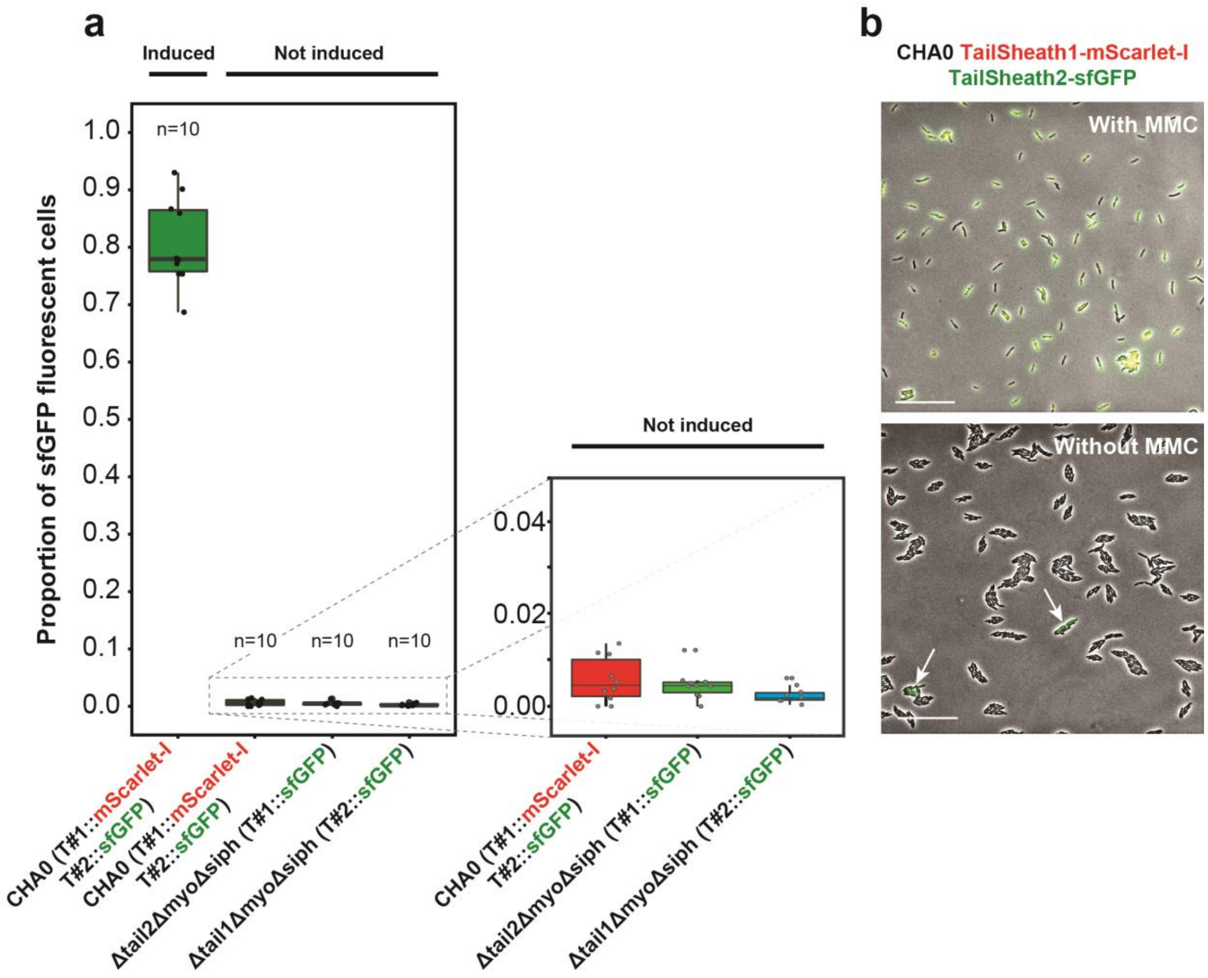

Supplementary Fig. 3. Mitomycin C (MMC) increases the induction of tailocins more than 80-fold. *Pseudomonas protegens* CHA0 TailSheath1-mScarlet-I TailSheath2-sfGFP (both tailocins tagged) cells were induced with MMC or not induced to calculate the proportions of induced cells (**a**). To induce cells, 3 mg mL^{-1} of MMC was added to an exponential growth culture for 30 min and then cells were washed prior to placing them under the microscope for 4 h. To verify that the deletion of the prophage background did not affect the production of tailocins, CHA0 Δtail2ΔmyoΔsiph (T#1::sfGFP), producing the tailocin #1 tagged with sfGFP, and CHA0 Δtail1ΔmyoΔsiph (T#2::sfGFP), producing the tailocin #2 tagged with sfGFP, were also tested in conditions without MMC. Snapshots of time-lapses either 3 h or 4 h post induction were analyzed with SuperSegger[3] using sfGFP as the induction signal. Ten positions were analyzed for each strain containing for CHA0 TailSheath1-mScarlet-I TailSheath2-sfGFP an average of 105 induced cells out of 130 (81 %) when induced with MMC and 5 induced cells out of 900 (0.61 %) without MMC. Positions for CHA0 Δtail2ΔmyoΔsiph T#1::sfGFP contained 4 induced cells out of 882 (0.59 %) and for CHA0 Δtail1ΔmyoΔsiph T#2::sfGFP 5 induced cells out of 2019 (0.26 %) without MMC). Individual results for each position are given in Supplementary Data 2. (**b**) Snapshot of CHA0 TailSheath1-mScarlet-I TailSheath2-sfGFP induced with MMC (top) and not induced with MMC (bottom). Arrows point out tailocin-producing cells in the condition without MMC. The scale bar represents 50 µm. To calculate the induction proportion, n=10 biological independent experiments were performed. The boxes indicate the interquartile range with the center representing the median.

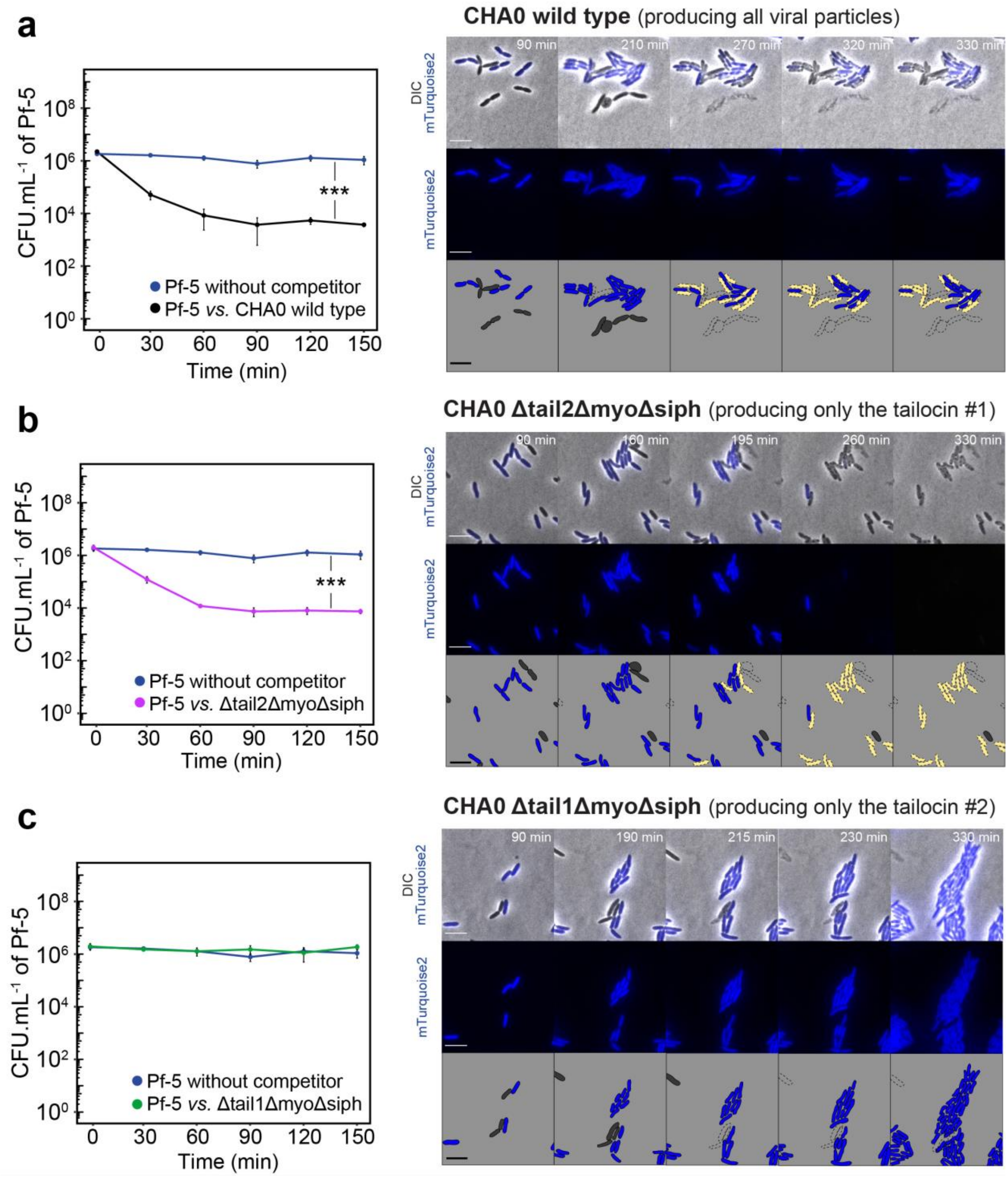

Supplementary Fig. 4. Tailocin #1 contributes to interbacterial competitiveness of *Pseudomonas protegens* CHA0 against *Pseudomonas protegens* Pf-5. mTurquoise2-tagged Pf-5 cells were faced with washed mitomycin C-induced cells of CHA0 wild type (**a**) and the CHA0 viral particle mutants, i.e. Δtail2ΔmyoΔsiph (**b**) or Δtail1ΔmyoΔsiph (**c**) in competition assays in liquid culture (left panels) and visualized by epifluorescence time-lapse microscopy (right panels). Cartoons outline the interaction at the different time points. CHA0 and Pf-5 mTurquoise2 live cells are shown in grey and blue respectively. Ghost cells are outlined with a dotted line and are either not colored (CHA0) or filled with yellow (Pf-5). Competition experiments in liquid culture were performed three times independently. Student's t-test was used to detect significant differences between the control condition and the other conditions at each time point (****P* value < 0.001). Error bars correspond to the standard deviation.

For the Pf-5 survival assay, n=3 biological independent experiments were performed. Error-bars correspond to the standard deviation.

Supplementary Tables

Supplementary Table 1. *Pseudomonas chlororaphis* and *Pseudomonas protegens* subgroup strains used in this study.

Strain [1]	Genome accession no.	Origin	Reference or source
***Pseudomonas chlororaphis* subgroup**			
P. chlororaphis O6	NZ_CM001490.1	Soil	[4]
P. chlororaphis R47	CP019399.1	Potato rhizosphere	[5]
P. chlororaphis subsp. *aureofaciens* 30-84	NZ_CM001559.1	Wheat rhizosphere	[4,6]
P. chlororaphis subsp. *aurantiaca* JD37	NZ_CP009290.1	Potato rhizosphere	[7,8]
P. chlororaphis subsp. *aureofaciens* CD	NZ_LHVB00000000.1	Cyclops (water)	[9,10]
P. chlororaphis subsp. *aureofaciens* LMG1245^T	NZ_LHVA00000000.1	River clay	[10,11]
P. chlororaphis subsp. *chlororaphis* LMG5004^T	NZ_LHVC00000000.1	Contaminated plate	[10,12]
P. chlororaphis subsp. *piscium* DSM21509^T (JF3835^T)	NZ_CP027707.1	European perch intestine	[13]
P. chlororaphis subsp. *piscium* PCL1391	NZ_CP027736.1	Tomato root	[10,14]
***Pseudomonas protegens* subgroup**			
P. protegens BRIP	NZ_LHUW00000000.1	Cyclops	[9,10]
P. protegens Cab57	NZ_AP014522.1	Shepperd's purse rhizosphere	[15]
P. protegens CHA0^T	LS999205.1	Tobacco rhizosphere	[16,17]
P. protegens K94.41	NZ_LHUU00000000.1	Cucumber rhizosphere	[10,18]
P. protegens PF	NZ_LHUX00000000.1	Wheat leaves	[10,19]
P. protegens Pf1	ERS3935804	Tobacco	[19], this study
P. protegens Pf-5	NC_004129.6	Cotton rhizosphere	[20,21]
P. protegens PGNR1	NZ_LHUV00000000.1	Tobacco rhizosphere	[10,19]
Pseudomonas sp. AU11706	NZ_LCZB00000000.1	Cystic fibrosis sputum	[22]
Pseudomonas sp. AU20219	NZ_LDET00000000.1	Cystic fibrosis sputum	[22]
Pseudomonas sp. AU13852	NZ_LCZC00000000.1	Cystic fibrosis sputum	[22]
Pseudomonas sp. CMR5c	NZ_LHUY00000000.1	Red cocoyam rhizosphere	[10,23]
Pseudomonas sp. CMR12a	CP027706.1	Red cocoyam rhizosphere	[10,23]
Pseudomonas sp. LD120	ERR3588830	Blade of marine alga	[24],[25]
Pseudomonas sp. Os17	NZ_AP014627.1	Rice rhizosphere	[15]
Pseudomonas sp. St29	NZ_AP014628.1	Potato rhizosphere	[15]

[1] ^{T}, type strain

Supplementary Table 2. *Pseudomonas protegens* CHA0 derivatives and *Escherichia coli* strains used in this study.

Strain name	Strain code	Genotype or relevant characteristics[1]	Reference or source
Pseudomonas protegens **CHA0 and derivatives**			
CHA0	$CHA0^T$	*P. protegens* type strain; wild type; genome accession no. LS999205.1	[16,17]
CHA0-*gfp2*	CHA0-*gfp2*	CHA0::attTn7-*gfp2*; Gm^R	[26]
Δtailcluster	CHA5285	Deletion of the entire R-tailocin gene cluster of CHA0 [from PPRCHA0_1217 to PPRCHA0_1252]	This study
Δtail1	CHA5287	Deletion of the sheath and tube genes of the tailocin #1 of CHA0 [PPRCHA0_1228 and PPRCHA0_1229]	This study
Δtail2	CHA5288	Deletion of the sheath and tube genes of the tailocin #2 of CHA0 [PPRCHA0_1238 and PPRCHA0_1239]	This study
Δmyo	CHA5297	Deletion of the entire *Myoviridae* prophage of CHA0 [from PPRCHA0_2016 to PPRCHA0_2057]	This study
ΔtailclusterΔmyo	CHA5289	CHA5285 with the deletion of the *Myoviridae* prophage	This study
ΔtailclusterΔsiph	CHA5292	CHA5285 with the deletion of the *Siphoviridae* prophage [from PPRCHA0_3761.1 to PPRCHA0_3804]	This study
Δtail1Δmyo	CHA5290	CHA5287 with the deletion of the *Myoviridae* prophage	This study
Δtail2Δsiph	CHA5298	CHA5288 with the deletion of the *Siphoviridae* prophage	This study
ΔmyoΔsiph	CHA5299	CHA0 with the deletion of the *Myoviridae* and *Siphoviridae* prophages	This study
ΔtailclusterΔsiphΔmyo	CHA5300	CHA0 with the deletion of the entire R-tailocin cluster and the *Myoviridae* and *Siphoviridae* prophages	This study
Δtail1ΔmyoΔsiph	CHA5301	CHA0 with the deletion of the tailocin #1 and the *Myoviridae* and *Siphoviridae* prophages	This study
Δtail2ΔmyoΔsiph	CHA5302	CHA0 with the deletion of the tailocin #2 and the *Myoviridae* and *Siphoviridae* prophages	This study
Δtail1Δtail2ΔmyoΔsiph	CHA5326	CHA0 with the deletion of the sheath and tube genes of the tailocins #1 and #2 and of the *Myoviridae* and *Siphoviridae* prophages	This study
Tailsheath1-mScarlet-I	CHA5294	CHA0::*tailsheath1-mscarlet-I*; CHA0 derivative expressing a C-terminal TailSheath1-mScarlet-I fusion protein	This study
Tailsheath2-sfGFP	CHA5295	CHA0::*tailsheath2-sfgfp*; CHA0 derivative expressing a C-terminal TailSheath2-sfGFP fusion protein	This study
TailSheath1-mScarlet-I TailSheath2-sfGFP	CHA5296	CHA0::*tailsheath1-mscarlet-I* and ::*tailsheath2-sfgfp*; CHA0 derivative expressing C-terminal TailSheath1-mScarlet-I and TailSheath2-sfGFP fusion proteins	This study
Tailsheath1-sfGFP Δtail2ΔmyoΔsiph	CHA5305	CHA5302::*tailsheath1-sfgfp*; CHA0 derivative with the deletion of the tailocin #2, the *Myoviridae* and *Siphoviridae* prophages, and expressing a C-terminal TailSheath1-sfGFP fusion protein	This study
Tailsheath2-sfGFP Δtail1ΔmyoΔsiph	CHA5306	CHA5301::*tailsheath2-sfgfp*; CHA0 derivative with the deletion of the tailocin #1, the *Myoviridae* and	This study

		Siphoviridae prophages, and expressing a C-terminal TailSheath2-sfGFP fusion protein	
***Pseudomonas protegens* Pf-5 and derivatives**			
Pf-5	Pf-5	Wild type; genome accession no. LS999205.1	19,20
Pf-5-mTurquoise2	Pf-5 mTurquoise2	Pf-5 strain tagged with miniTn*7*::*mturquoise2*; Gm^R	This study
Escherichia coli			
E. coli S17-1/λpir		Laboratory strain	[27]
E. coli DH5α		Laboratory strain	[28]

[1] Gm^R, gentamicin resistance.

Supplementary Table 3. Inventory of the viral particles present in the *Pseudomonas protegens* CHA0 mutant strains.

Strain name [1]	Strain code	Viral particles present			
		Tailocin #1	Tailocin #2	Myovirus	Siphovirus
CHA0	CHA0^{T}	X	X	X	X
Δtailcluster	CHA5285			X	X
Δtail1	CHA5287		X	X	X
Δtail2	CHA5288	X		X	X
Δmyo	CHA5297	X	X		X
ΔtailclusterΔmyo	CHA5289				X
ΔtailclusterΔsiph	CHA5292			X	
Δtail1Δmyo	CHA5290		X		X
Δtail2Δsiph	CHA5298	X		X	
ΔmyoΔsiph	CHA5299	X	X		
ΔtailclusterΔsiphΔmyo	CHA5300				
Δtail1ΔmyoΔsiph	CHA5301		X		
Δtail2ΔmyoΔsiph	CHA5302	X			

[1] See Supplementary Table 2 for detailed information on the strain genotype.

Supplementary Table 4. Details of the tailocins #1 and #2 encoding genomic region in *Pseudomonas protegens* CHA0.

Gene name	Locus tag [1]	Predicted function [2]
mutS	PPRCHA0_1216	DNA mismatch repair protein MutS
prtR1	PPRCHA0_1217	Helix-turn-helix transcriptional regulator; related to the pyocin repressor PrtR of *Pseudomonas aeruginosa* (41.6% amino acid identity)
hol	PPRCHA0_1218	Holin
	PPRCHA0_1219	Phage baseplate/tail assembly protein U; putative tail tube initiator
	PPRCHA0_1220	Phage baseplate assembly protein V; tail spike
	PPRCHA0_1221.5	Phage baseplate assembly protein W, wedge subunit; GPW_gp25 superfamily; manually updated annotation that regroups PPRCHA0_1221 and PPRCHA0_1222
	PPRCHA0_1223	Phage baseplate assembly protein J
	PPRCHA0_1224	Phage tail protein I; phage baseplate/tail assembly
	PPRCHA0_1225	Phage-related tail fiber protein
	PPRCHA0_1226	Putative tail fiber charperone
	PPRCHA0_1227	Putative tail assembly chaperone
	PPRCHA0_1228	Phage tail sheath protein
	PPRCHA0_1229	Phage major tail tube protein
	PPRCHA0_1230	Phage tail assembly chaperone
	PPRCHA0_1231	Phage tail tape measure protein
	PPRCHA0_1232	Phage baseplate/tail assembly protein U; putative tail tube initiator
	PPRCHA0_1233	Phage tail protein X; predicted peptidoglycan binding
	PPRCHA0_1234	Phage late control gene D protein; baseplate/tail assembly
	PPRCHA0_1235	Putative endolysin; peptidoglycan hydrolase; glycosyl hydrolases family 18 protein
	PPRCHA0_1236	Putative tail completion protein
	PPRCHA0_1237	Phage protein with DUF2635 domain; putative tail sheath termination and capping
	PPRCHA0_1238	Phage tail sheath protein
	PPRCHA0_1239	Phage major tail tube protein
	PPRCHA0_1240	Phage tail assembly chaperone
	PPRCHA0_1241	Phage tail tape measure protein
	PPRCHA0_1242	Possible hub between the tail and the tail-tip/baseplate; contains DNA circularization domain
	PPRCHA0_1243	Phage late control gene D protein; baseplate/tail assembly
	PPRCHA0_1244	Phage baseplate assembly protein V; tail spike
	PPRCHA0_1245	Phage baseplate assembly protein W, wedge subunit; GPW_gp25 superfamily
	PPRCHA0_1246	Phage baseplate assembly protein J
	PPRCHA0_1247	Putative phage tail protein; contains DUF2313 domain
	PPRCHA0_1248	Phage tail fiber protein
	PPRCHA0_1249	Putative tail assembly chaperone
	PPRCHA0_1250	Putative endolysin; peptidoglycan hydrolase; glycoside hydrolase family 19 protein
	PPRCHA0_1251<br>PPRCHA0_1251.1	Putative spanin organized in Russian dolls (with manually annotated PPRCHA0_1251.1)
	PPRCHA0_1252	Unknown function
cinA	PPRCHA0_1253	Competence/damage-inducible protein CinA
recA	PPRCHA0_1254	Bacterial DNA recombination protein RecA

[1] Genome accession number LS999205.1. Dotted horizontal lines and grey shading delimit the two clusters predicted to be required for assembly of tailocin #1 (upper box) and tailocin #2 (lower box), respectively. Lysis and regulation functions are highlighted with light yellow and light blue shading, respectively.
[2] Functions were predicted based on characterization of proteins encoded by the R-tailocins gene cluster with NCBI Conserved Domain Database Search[29] and Phyre2[30].

Supplementary Table 5. Measurement of the length of CHA0 R-tailocins.

**Tailocin type**	**Length (nm)**	**Number of particles measured**
Tailocin #1 non-contracted	157.7 ± 11.0	34
Tailocin #1 contracted	64.9 ± 11.6	9
Tailocin #1 tagged with mScarlet-I	101.9 ± 5.8	45
Tailocin #2 non-contracted	100.7 ± 5.7	20
Tailocin #2 contracted	56.3 ± 4.9	40
Tailocin #2 tagged with sfGFP	104.7 ± 6.0	21

Supplementary Table 6. Number of *Pseudomonas protegens* Pf-5 cells killed by either *Pseudomonas protegens* CHA0 wild type or CHA0 ΔtailΔ2myoΔsiph cells in non-induced conditions.

	Biological replicate	Position on slide	Number of CHA0 lysed that killed Pf-5	Number of Pf-5 killed by CHA0	Average number of Pf-5 killed by CHA0	Total number of biological replicates	Total number of observed events per total position analyzed
CHA0 wild-type *vs.* Pf-5	1	#5	2	47	17.62	4	8/21
		#6	1	20			
		#7	2	58			
	2	#14	1	5			
	3	#1	2	46			
		#2	2	19			
		#3	1	9			
		#11	2	25			
CHA0 Δtail2myoΔsiph vs. Pf-5	3	#9	2	32	12.64	4	6/30
		#16	2	11			
	4	#7	1	23			
		#8	1	21			
		#9	3	45			
		#16	2	7			

Supplementary Table 7. Plasmids used in this study.

**Plasmid**	**Genotype or relevant characteristics**[1]	**Reference or source**
pBK-miniTn7-*gfp2*	pUC19-based delivery plasmid for miniTn7-*gfp2*; mob^{+}; Gm^{R}, Cm^{R}, Ap^{R}	[31]
pBK-miniTn7-mTurquoise2	pUC18T-mini-Tn7T-Gm-Pc-mTurquoise2	[32]
pEMG	Expression vector; *ori*R6K, *lacZα* with two flanking I-SceI sites; Km^{R}, Ap^{R}	1
pME11079	pEMG::*Δtailcluster*; suicide plasmid for the deletion of the entire R-tailocin gene cluster; Km^{R}	This study
pME11080	pEMG::*Δtail2*; suicide plasmid for the deletion of the tailocin #2 sheath and tube genes; Km^{R}	This study
pME11081	pEMG::*Δtail1*; suicide plasmid for the deletion of the tailocin #1 sheath and tube genes *Δtail1*; Km^{R}	This study
pME11082	pEMG::*Δsiph*; suicide plasmid for the deletion of the entire *Siphoviridae* prophage; Km^{R}	This study
pME11085	pEMG::*Δmyo*; suicide plasmid for the deletion of the entire *Myoviridae* prophage; Km^{R}	This study
pME11087	pEMG::*tailsheath1-mscarlet-I;* suicide plasmid for 3' tagging of the sheath protein of the tailocin #1 with mScarlet-I; Km^{R}	This study
pME11088	pEMG::*tailsheath2-sfgfp;* suicide plasmid for 3' tagging of the sheath protein of the tailocin #2 with sfGFP; Km^{R}	This study
pME497	Mobilizing plasmid; IncP-1 Tra RepA (Ts); Ap^{R}	[33]

[1] Ap^{R}, ampicillin resistance; Cm^{R}, chloramphenicol resistance; Gm^{R}, gentamycin resistance; Km^{R}, kanamycin resistance; Tc^{R}, tetracycline resistance.

Supplementary Table 8. Oligonucleotides used in this study.

Primer	Sequence (5'-3') [1]	Application
Tail-1	CGGAATTCATCAGCATCCTCGGTCACGTC	Deletion of CHA0 entire tailocin #1 and #2 gene cluster
Tail-2	GGGGTACCTGCGGATTGAGGCCAGCGAAAT	Deletion of CHA0 entire tailocin #1 and #2 gene cluster
Tail-3	GGGGTACCTCTGGAGGCCGCGCGTGATTG	Deletion of CHA0 entire tailocin #1 and #2 gene cluster
Tail-4	ACGCGTCGACGGCGGACCTCATCGCGATTTC	Deletion of CHA0 entire tailocin #1 and #2 gene cluster
Check alltail-F	ATTCTCCCAGCCCTTGATTT	Verification of *Δtailcluster* mutant of CHA0
Check alltail-R	CTACCCCTGATTTGCGATTT	Verification of *Δtailcluster* mutant of CHA0
SheathTube1-1	CGGAATTCAAAGTTCTCTCTGTGTCGGCG	Deletion of CHA0 tube and sheath genes of tailocin #1
SheathTube1-2	CGGGATCCACTCATGGATAAACTCCAGAC	Deletion of CHA0 tube and sheath genes of tailocin #1
SheathTube1-3	CGGGATCCGGCCTGTAAGGAAAAAACATG	Deletion of CHA0 tube and sheath genes of tailocin #1
SheathTube1-4	ACGCGTCGACCTTTTTCCATTCATCCTGCAG	Deletion of CHA0 tube and sheath genes of tailocin #1
Check Tail1-F	CTGGCGGTTATTCGATTGAG	Verification of *Δtail1* mutant of CHA0
Check Tail1-R	GCCTGATATTCCTGACGCAG	Verification of *Δtail1* mutant of CHA0
SheathTube2-1	CGGAATTCTACAACCCGATCCAGTACGAC	Deletion of CHA0 tube and sheath genes of tailocin #2
SheathTube2-2	GGGGTACCCATTATTTGGCTCCTTGAGA	Deletion of CHA0 tube and sheath genes of tailocin #2
SheathTube2-3	GGGGTACCCAATGAGCAACACGGTGAAGC	Deletion of CHA0 tube and sheath genes of tailocin #2
SheathTube2-4	CGGGATCCGGTCTGCTCAAGGTTTTTCTT	Deletion of CHA0 tube and sheath genes of tailocin #2
Check Tail2-F	CATCATTCGCCAGACTATCC	Verification of *Δtail2* mutant of CHA0
Check Tail2-R	AGAAACGGGGTGATCAGG	Verification of *Δtail2* mutant of CHA0
Myo-1	GGGGTACCTTCGAGAATGGCGATTCGTG	Deletion of the entire *Myoviridae* prophageof CHA0
Myo-2	CGGGATCCCATATCGAAAGTGGTACGCG	Deletion of the entire *Myoviridae* prophage of CHA0
Myo-3	CGGGATCCGTAAGATCCGCAAAGTGAGT	Deletion of the entire *Myoviridae* prophage of CHA0
Myo-3	ACGCGTCGACAAACGTAGCGAAAAGATCGC	Deletion of the entire *Myoviridae* prophage of CHA0
Check Myo-F	GATGGTTAACAGGGAGGTGA	Verification of myovirus deletion mutant of CHA0
Check Myo-R	AAACTCTCTAAGCGCAGTCT	Verification of myovirus deletion mutant of CHA0
Siph-1	CGGAATTCATAACCGACACGCATCTG	Deletion of the entire *Siphoviridae* prophage of CHA0
Siph-2	CGGGATCCTAGCAACTGGGGGTGTTT	Deletion of the entire *Siphoviridae* prophage of CHA0
Siph-3	CGGGATCCCGCTGACAGACAATCGAA	Deletion of the entire *Siphoviridae* prophage of CHA0
Siph-4	ACGCGTCGACGCCAGCTTGTCCTCTGTT	Deletion of the entire *Siphoviridae* prophage of CHA0
Check Siph-F	GCCATTTCGTCGTAGAAGAC	Verification of siphovirus deletion mutant of CHA0
Check Siph-R	TCGAAAGCCGAAATCGTGAA	Verification of siphovirus deletion mutant of CHA0
Check-fus1-F1	TGTTTGCCTGGACCGACA	Verification of CHA0 TailSheath1-mScarlet-I
Check-fus1-R1	CTGTTACAGCTCCTCCGT	Verification of CHA0 TailSheath1-mScarlet-I
Check-fus1-F2	AAAGGACGGAGGCCGATA	Verification of CHA0 TailSheath1-mScarlet-I
Check-fus1-R2	GGGCATCGATCTCGTAGA	Verification of CHA0 TailSheath1-mScarlet-I
Check-fus2-F1	GCGGACTTCGGTGTTCAT	Verification of CHA0 TailSheath2-sfGFP
Check-fus2-R1	CGTCTTGTAGGTCCCGTC	Verification of CHA0 TailSheath2-sfGFP
Check-fus2-F2	CGTGACCACATGGTCCTT	Verification of CHA0 TailSheath2-sfGFP
Check-fus2-R2	CTTGATCGCCCGTACTTC	Verification of CHA0 TailSheath2-sfGFP
pEMG check-F	GTAAAACGACGGCCAGT	Sequencing verification of the pEMG-based plasmids
pEMG check-R	AACAGCTATGACCATG	Sequencing verification of the pEMG-based plasmids

[1] Restriction sites are underlined.

Supplementary References

1. Martínez-García, E. & de Lorenzo, V. Engineering multiple genomic deletions in Gram-negative bacteria: analysis of the multi-resistant antibiotic profile of *Pseudomonas putida* KT2440. *Environ. Microbiol.* **13**, 2702–2716 (2011).

2. Kupferschmied, P., Péchy-Tarr, M., Imperiali, N., Maurhofer, M. & Keel, C. Domain shuffling in a sensor protein contributed to the evolution of insect pathogenicity in plant-beneficial *Pseudomonas protegens*. *PLoS Pathog.* **10**, e1003964 (2014).

3. Stylianidou, S., Brennan, C., Nissen, S. B., Kuwada, N. J. & Wiggins, P. A. SuperSegger: robust image segmentation, analysis and lineage tracking of bacterial cells. *Mol. Microbiol.* **102**, 690–700 (2016).

4. Loper, J. E. *et al.* Comparative genomics of plant-associated *Pseudomonas* spp.: insights into diversity and inheritance of traits involved in multitrophic interactions. *PLoS Genet.* **8**, e1002784 (2012).

5. De Vrieze, M. *et al.* Volatile organic compounds from native potato-associated *Pseudomonas* as potential anti-oomycete agents. *Front. Microbiol.* **6**, (2015).

6. Pierson, L. S. & Thomashow, L. S. Cloning and heterologous expression of the phenazine biosynthetic locus from *Pseudomonas aureofaciens* 30-84. *Mol. Plant-Microbe Interact.* **5**, 330–339 (1992).

7. Jiang, Q. *et al.* Complete genome sequence of the plant growth-promoting rhizobacterium *Pseudomonas aurantiaca* strain JD37. *J. Biotechnol.* **192 Pt A**, 85–86 (2014).

8. Fang, R. *et al.* Promotion of plant growth, biological control and induced systemic resistance in maize by *Pseudomonas aurantiaca* JD37. *Ann. Microbiol.* **63**, 1177–1185 (2013).

9. Ruffner, B. *et al.* Evolutionary patchwork of an insecticidal toxin shared between plant-associated pseudomonads and the insect pathogens *Photorhabdus* and *Xenorhabdus*. *BMC Genomics* **16**, 609 (2015).

10. Flury, P. *et al.* Insect pathogenicity in plant-beneficial pseudomonads: phylogenetic distribution and comparative genomics. *ISME J.* **10**, 2527–2542 (2016).

11. Kluyver, A. J. *Pseudomonas aureofaciens* nov. spec. and its pigments. *J. Bacteriol.* **72**, 406–411 (1956).

12. Peix, A. *et al.* Reclassification of *Pseudomonas aurantiaca* as a synonym of *Pseudomonas chlororaphis* and proposal of three subspecies, *P. chlororaphis* subsp. *chlororaphis* subsp. nov., *P. chlororaphis* subsp. *aureofaciens* subsp. nov., comb. nov. and *P. chlororaphis* subsp. *aurantiaca* subsp. nov., comb. nov. *Int. J. Syst. Evol. Microbiol.* **57**, 1286–1290 (2007).

13. Burr, S. E., Gobeli, S., Kuhnert, P., Goldschmidt-Clermont, E. & Frey, J. *Pseudomonas chlororaphis* subsp. *piscium* subsp. nov., isolated from freshwater fish. *Int. J. Syst. Evol. Microbiol.* **60**, 2753–2757 (2010).

14. Chin-A-Woeng, T. F. C. *et al.* Biocontrol by phenazine-1-carboxamide-producing *Pseudomonas chlororaphis* PCL1391 of tomato root rot caused by *Fusarium oxysporum* f. sp. *radicis-lycopersici*. *Mol. Plant Microbe Interact.* **11**, 1069–1077 (1998).

15. Takeuchi, K. *et al.* Rhizoxin analogs contribute to the biocontrol activity of newly isolated *Pseudomonas* strain. *Mol. Plant-Microbe Interact.* **28**, 333-342 (2014).

16. Stutz, E. W., Défago, G. & Kern, H. Naturally occurring fluorescent pseudomonads involved in suppression of black root rot of tobacco. *Phytopathology* **76**, 181–185 (1986).

17. Smits, T. H. M. *et al.* Updated genome sequence and annotation for the full genome of *Pseudomonas protegens* CHA0. *Microbiol. Res. Announc.* (2019).

18. Wang, C. *et al.* Cosmopolitan distribution of *phlD*-containing dicotyledonous crop-associated biocontrol pseudomonads of worldwide origin. *FEMS Microbiol. Ecol.* **37**, 105–116 (2001).

19. Keel, C. *et al.* Conservation of the 2,4-diacetylphloroglucinol biosynthesis locus among fluorescent *Pseudomonas* strains from diverse geographic locations. *Appl. Environ. Microbiol.* **62**, 552–563 (1996).

20. Howell, C. R. & Stipanovic, R. D. Control of *Rhizoctonia solani* on cotton seedlings with *Pseudomonas fluorescens* and with an antibiotic produced by the bacterium. *Phytopathology* **69**, 480–482 (1979).

21. Paulsen, I. T. *et al.* Complete genome sequence of the plant commensal *Pseudomonas fluorescens* Pf-5. *Nat. Biotechnol.* **23**, 873–878 (2005).

22. Scales, B. S., Erb-Downward, J. R., LiPuma, J. J. & Huffnagle, G. B. Draft genome sequences of five *Pseudomonas fluorescens* subclade I and II strains, isolated from human respiratory samples. *Genome Announc.* **3**, e00837-15 (2015).

23. Perneel, M. *et al.* Characterization of CMR5c and CMR12a, novel fluorescent *Pseudomonas* strains from the cocoyam rhizosphere with biocontrol activity. *J. Appl. Microbiol.* **103**, 1007–1020 (2007).

24. Nagel, K. *et al.* Beneficial effects of 2,4-diacetylphloroglucinol-producing pseudomonads on the marine alga *Saccharina latissima. Aquat. Microb. Ecol.* **67**, 239–249 (2012).

25. Heiman, C. M. *et al.* Draft genome sequence of *Pseudomonas* sp. LD120 isolated from the marine alga *Saccharina latissima*. *Microbiol. Resour. Announc.* 01305-19 (2020).

26. Marchler-Bauer, A. *et al.* CDD/SPARCLE: functional classification of proteins via subfamily domain architectures. *Nucleic Acids Res.* **45**, D200–D203 (2017).

27. Kelley, L. A., Mezulis, S., Yates, C. M., Wass, M. N. & Sternberg, M. J. E. The Phyre2 web portal for protein modeling, prediction and analysis. *Nat. Protoc.* **10**, 845–858 (2015).

28. Péchy-Tarr, M. *et al.* Control and host-dependent activation of insect toxin expression in a root-associated biocontrol pseudomonad. *Environ. Microbiol.* **3**, 732-750 (2013).

29. Simon, R., Priefer, U. & Pühler, A. A broad host range mobilization system for *in vivo* genetic engineering: Transposon mutagenesis in gram negative bacteria. *Nat. Biotechnol.* **1**, 784–791 (1983).

30. Sambrook, J., Fritsch, E. F. & Maniatis, T. Molecular cloning: a laboratory manual. *Molecular cloning: a laboratory manual.* (1989).

31. Koch, B., Jensen, L. E. & Nybroe, O. A panel of Tn7-based vectors for insertion of the *gfp* marker gene or for delivery of cloned DNA into Gram-negative bacteria at a neutral chromosomal site. *J. Microbiol. Methods.* **45**, 187–195 (2001).

32. Wilton, R. *et al.* A new suite of plasmid vectors for fluorescence-based imaging of root colonizing pseudomonads. *Front. Plant Sci.* **8**, 2242 (2018).

33. Schnider-Keel, U. *et al.* Autoinduction of 2,4-diacetylphloroglucinol biosynthesis in the biocontrol agent *Pseudomonas fluorescens* CHA0 and repression by the bacterial metabolites salicylate and pyoluteorin. *J. Bacteriol.* **182**, 1215–1225 (2000).
