# Supplementary material for: Live cell dynamics of production, explosive release and killing activity of phage tail-like weapons for Pseudomonas kin exclusion
Source: Commun Biol. 2021 Jan 19;4:87. doi: 10.1038/s42003-020-01581-1 (PMC7815802; doi:10.1038/s42003-020-01581-1)
Supplement: Supplementary file 3 — Description of additional supplementary files [file 42003_2020_1581_MOESM3_ESM.pdf]

Description of Additional Supplementary Files

Supplementary Movie 1. Production dynamic of the R-tailocins of Pseudomonas protegens CHA0 following induction. The sheath subunit proteins of the tailocins #1 and #2 were tagged with mScarlet-I (magenta) and sfGFP (green), respectively. Cells were monitored post induction with mitomycin C using time-lapse microscopy (time post induction shown in upper right corner). White arrows follow the migration of the R-tailocins from the cell center to the poles of the cell prior to cell lysis. The scale bar represents 5 µm. From left to right: bright field, mCherry and GFP channels are shown.

Supplementary Movie 2. Production dynamic of the R-tailocins of Pseudomonas protegens CHA0 following induction - Migration to cell poles. The sheath subunit proteins of the tailocins #1 and #2 were tagged with mScarlet-I (magenta) and sfGFP (green), respectively. Cells were monitored post induction with mitomycin C using time-lapse microscopy (time post induction shown in upper right corner). White arrows follow the migration of the R-tailocins from the cell center to the poles of the cell prior to cell lysis. The scale bar represents 5 µm. From left to right: bright field, mCherry and GFP channels are shown.

Supplementary Movie 3. Production dynamic of the R-tailocins of Pseudomonas protegens CHA0 following induction - The burst. The sheath subunit proteins of the tailocins #1 and #2 were tagged with mScarlet-I (magenta) and sfGFP (green), respectively. Cells were monitored post induction with mitomycin C using time-lapse microscopy (time post induction shown in upper right corner). White arrows indicate the cells that undergo lysis. Cells lyse and thrust the produced R-tailocins at 124 min and 140 min. The scale bar represents 5 µm. R-tailocins are projected at several tens of micrometers from the producing cell. From left to right: bright field, mCherry and GFP channels are shown. The brightness was artificially enhanced to better visualize the fluorescent R-tailocins in the medium.

Supplementary Movie 4. Competition between induced Pseudomonas protegens CHA0 with fluorescent R-tailocins and Pseudomonas protegens Pf-5. Induced CHA0 cells produce R-tailocins at the center of the cell and the particles then migrate to the poles prior to cell lysis. Once R-tailocins are thrusted into the environment, they can reach competing Pf-5 mTurquoise2 tagged cells. White arrows indicate the lysis of Pf-5 cells. CHA0 cells with tailocins #1 and #2, respectively tagged with mScarlet-I (magenta) and sfGFP (green), were induced with mitomycin C, washed and confronted with Pf-5 mTurquoise2 cells and monitored with epifluorescence time-lapse microscopy. The time post induction is shown in the upper right corner. The scale bar represents 5 µm. From left to right: bright field, mCherry, GFP and CFP channels are shown.

Supplementary Movie 5. Competition between induced Pseudomonas protegens CHA0 with fluorescent R-tailocins and Pseudomonas protegens Pf-5. Induced CHA0 cells produce R-tailocins at the center of the cell and the particles then migrate to the poles prior to cell lysis. Once R-tailocins are thrusted into the environment, they can reach competing Pf-5 mTurquoise2 tagged cells. White arrows indicate the lysis of Pf-5 cells. CHA0 cells with tailocins #1 and #2, respectively tagged with mScarlet-I (magenta) and sfGFP (green), were induced with mitomycin C, washed and confronted with Pf-5 mTurquoise2 cells and monitored with epifluorescence

time-lapse microscopy. The time post induction is shown in the upper right corner. The scale bar represents 5 µm. From left to right: bright field, mCherry, GFP and CFP channels are shown.

Supplementary Movie 6. Competition between Pseudomonas protegens CHA0 wild type and Pseudomonas protegens Pf-5 (non-induced). The competition between Pf-5 mTurquoise2 and CHA0 wild type was followed by time-lapse microscopy. In this experiment, CHA0 was not artificially induced with mitomycin C (non-induced conditions). Following the lysis and the assumed production of Rtailocins, Pf-5 cells are killed. White arrows indicate the lysis of CHA0 cells following the production of Rtailocins (bright field, left) as well as the lysis of Pf-5 cells (CFP, right). The time post contact is shown in the upper right corner. The scale bar represents 5 µm. From left to right: bright field and CFP channels are shown.

Supplementary Movie 7. Competition between Pseudomonas protegens CHA0 Δtail2ΔmyoΔsiph and Pseudomonas protegens Pf-5 (non-induced). The competition between Pf-5 mTurquoise2 and CHA0 Δtail2ΔmyoΔsiph was followed by time-lapse microscopy. CHA0 was not artificially induced with mitomycin C in this assay (non-induced conditions). Following the lysis and the assumed production and release of the tailocin #1 particles, Pf-5 cells are killed. White arrows indicate the lysis of CHA0 cells following the production of R-tailocins (bright field, left) as well as the lysis of P. protegens Pf-5 cells (CFP, right). The time post contact is shown in the upper right corner. The scale bar represents 5 µm. From left to right: bright field and CFP channels are shown.

Supplementary Movie 8. Competition between Pseudomonas protegens CHA0 Δtail1ΔmyoΔsiph and Pseudomonas protegens Pf-5 (non-induced). The competition between P. protegens Pf-5 mTurquoise2 and P. protegens CHA0 Δtail1ΔmyoΔsiph was followed by time-lapse microscopy. CHA0 was not artificially induced with mitomycin C in this assay (non-induced conditions). Although P. protegens CHA0 lyses and assumedly produces tailocin #2, Pf-5 cells are not affected. This demonstrates that the released tailocin #2 particles have no effect on the survival of Pf-5 cells. White arrows indicate the lysis of CHA0 cells. The time post contact is shown in the upper right corner. The scale bar represents 5 µm. From left to right: bright field and CFP channels are shown.

Supplementary Movie 9. Competition between Pseudomonas protegens CHA0 wild type induced with mitomycin C and Pseudomonas protegens Pf-5. The competition between Pf-5 mTurquoise2 and induced CHA0 wild type was followed by time-lapse microscopy. Following the lysis and the assumed release of R-tailocins, Pf-5 cells are killed. White arrows indicate the lysis of CHA0 cells following the production of R-tailocins (bright field, left). The time post induction is shown in the upper right corner. The scale bar represents 5 µm. From left to right: bright field, and CFP channels are shown.

Supplementary Movie 10. Competition between Pseudomonas protegens CHA0 Δtail2ΔmyoΔsiph induced with mitomycin C and Pseudomonas protegens Pf-5. The competition between Pf-5 mTurquoise2 and induced CHA0 Δtail2ΔmyoΔsiph was followed by time-lapse microscopy. Following the lysis and the assumed production and release of the tailocin #1 particles, Pf-5 cells are killed. White arrows indicate the lysis of CHA0 cells following the production of R-tailocins (bright field, left). The time post induction is shown in the upper right

corner. The scale bar represents 5 µm. From left to right: bright field, and CFP channels are shown.

Supplementary Movie 11. Competition between Pseudomonas protegens CHA0 Δtail1ΔmyoΔsiph induced with mitomycin C and Pseudomonas protegens Pf-5. The competition between Pf-5 mTurquoise2 and induced CHA0 Δtail1ΔmyoΔsiph was followed by time-lapse microscopy. Although CHA0 lyses and assumedly produces tailocin #2, Pf-5 cells are not affected. This demonstrates that the released tailocin #2 particles have no effect on the survival of Pf-5 cells. White arrows indicate the lysis of CHA0 cells. The time post induction is shown in the upper right corner. The scale bar represents 5 µm. From left to right: bright field, and CFP channels are shown.

Supplementary Movie 12. Long-range effect of tailocin #1 of Pseudomonas protegens CHA0 Δtail2ΔmyoΔsiph on Pseudomonas protegens Pf-5. The competition between Pf-5 mTurquoise2 and induced CHA0 Δtail2ΔmyoΔsiph was followed by time-lapse microscopy. CHA0 lyses and assumedly produces tailocin #1 that targets Pf-5 cells that are not in the vicinity of the producing CHA0. This demonstrates that the released tailocin #1 particles are thrusted in the environment and can target and kill distant kin competitors. The time post induction is shown in the upper right corner. The scale bar represents 5 µm. From left to right: bright field, and CFP channels are shown.

Supplementary Data 1. Dilution assay of purified viral particles on different Pseudomonas strains

Supplementary Data 2. Mitomycin C (MMC) increases the induction of tailocins more than 80-fold. Pseudomonas protegens CHA0 TailSheath1-mScarlet-I TailSheath2-sfGFP (both tailocins tagged) cells were induced with MMC or not induced to calculate the proportions of induced cells.

Supplementary Data 3. Competition assays.
